# Blocking the Wnt/β-Catenin Pathway by Lentivirus-Mediated Short Hairpin RNA Targeting β-Catenin Gene Suppresses Silica-Induced Lung Fibrosis in Mice

**DOI:** 10.3390/ijerph120910739

**Published:** 2015-09-01

**Authors:** Xin Wang, Wujing Dai, Yanrang Wang, Qing Gu, Deyi Yang, Ming Zhang

**Affiliations:** 1 Tianjin Center for Disease Control and Prevention, Tianjin 300011, China; E-Mails: h_angel@126.com (X.W.); yangdeyi2013@163.com (D.Y.); zhangming0124@yeah.net (M.Z.); 2 College of Public Health, Tianjin Medical University, Tianjin 300070, China; 3 Division of Pneumoconiosis, School of Public Health, China Medical University, Shenyang 110122, China; E-Mail: wujingDai2012@126.com

**Keywords:** silicosis, Wnt/β-catenin pathway, RNA interference, lentivirus, MMPs

## Abstract

Silicosis is a form of occupational lung disease caused by inhalation of crystalline silica dust. While the pathogenesis of silicosis is not clearly understood, the Wnt/β-catenin signaling pathway is thought to play a major role in lung fibrosis. To explore the role of Wnt/β-catenin pathway in silicosis, we blocked Wnt/β-catenin pathway both in silica-treated MLE-12 cells (a mouse pulmonary epithelial cell line) and in a mouse silicosis model by using a lentiviral vector expressing a short hairpin RNA silencing β-catenin (Lv-shβ-catenin). *In vitro*, Lv-shβ-catenin significantly decreased the expression of β-catenin, MMP2 and MMP9, and secretion of TGF-β1. *In vivo*, intratracheal treatment with Lv-shβ-catenin significantly reduced expression of β-catenin in the lung and levels of TGF-β1 in bronchoalveolar lavage fluid, and notably attenuated pulmonary fibrosis as evidenced by hydroxyproline content and collagen I\III synthesis in silica-administered mice. These results indicate that blockade of the Wnt/β-catenin pathway can prevent the development of silica-induced lung fibrosis. Thus Wnt/β-catenin pathway may be a target in prevention and treatment of silicosis.

## 1. Introduction

Silicosis is a form of occupational lung disease caused by inhalation of crystalline silica dust [1,2]. However, the pathogenesis of silicosis is not clearly understood. The Wnt/β-catenin signaling pathway participates in development, regeneration, and remodeling of the lung. Dysregulation of Wnt/β-catenin signaling results in various diseases of lung tissue, such as lung cancer and idiopathic pulmonary fibrosis [3,4]. Recent studies have suggested that the Wnt/β-catenin signaling pathway plays an important role in pulmonary fibrosis [5,6], which is a typical pathological characteristic in silicosis.

The Wnt signaling pathway is highly conserved in evolution. It can be divided into three distinct pathways, and the “canonical” β-catenin dependent Wnt signaling pathway is best characterized [7,8]. This pathway involves the regulation of cytosolic stabilization and nuclear translocation of β-catenin through binding of Wnt ligand to cell surface receptors [9]. β-Catenin is the most important effector molecule in this pathway. After lung tissue injury, abnormal activation of the Wnt/β-catenin signaling pathway could change expression, distribution and function of β-catenin in pulmonary epithelial cells. Activation of cytokine signaling and cell recruitment in the areas of silica dust deposition can lead to excess disruption of extracellular matrix, which is associated with the secretion of matrix metalloproteinases (MMPs) [10,11]. Dysregulation of β-catenin may result in up-regulation of the downstream genes, such as MMP2 and MMP9, which are known as molecular markers associated with fibrosis [10]. Moreover, injured alveolar epithelia release various cytokines including transforming growth factor-β1 (TGF-β1), which plays a critical role in the progression of lung fibrosis [12]. The release of TGF-β1 is also related to the Wnt/β-catenin signaling pathway [13].

In the present study, we generated a lentiviral vector expressing a short hairpin RNA (shRNA) that targeted β-catenin gene of mouse (Lv-shβ-catenin). A mouse pulmonary epithelial cell line MLE-12 and a mouse silicosis model were used to estimate the effect of β-catenin inhibition on silicosis. We observed that Lv-shβ-catenin decreased the expression of β-catenin, meanwhile the expression of MMP2, MMP9 and secretion of TGF-β1 both *in vitro* and *in vivo*. In addition, intratracheal of Lv-shβ-catenin suppressed β-catenin expression and attenuated lung fibrosis in the mouse silicosis model.

## 2. Methods and Material

### 2.1. Construction of the Lentivirus Vectors of β-Catenin

Five siRNA sequences were designed against the mouse β-catenin sequence in GenBank (NM_007614.3) using on-line siRNA software (http://ihome.ust.hk/~bokcmho/siRNA/siRNA.html). A negative control sequence cited by many other papers was chosen [14]. The shRNA expressing vectors were generated by inserting annealed oligo sequences into the digested pLKD-GFP vectors (Shanghai NeuronBiotech Co., Ltd., Shanghai, China) on the basis of the siRNA sequences described above, named pLKD-β-catenin -1, -2, -3, -4, -5, or non-silenced control lentivirus (NC), respectively. Details of the synthesized sequence are listed in Table 1. Lentiviruses were produced in HEK-293T cells (American Type Culture Collection (ATCC), Roskilde, Denmark) by transient transfection of three plasmids (Shanghai NeuronBiotech Co., Ltd., Shanghai, China): the transfer vector pLKD-β-catenin -1, -2, -3, -4,-5, or -NC, the packaging vector psPAX2 and the VSV-G expression plasmid pMD2G, followed by purification by ultracentrifugation, as previously described [15]. The lentivirus vector named as Lv-β-catenin-1, -2, -3, -4, -5, or -NC.

**Table 1:** The sequence of synthesized oligonucleotides.

Item Sequence
Shβ-catenin -1 Oligo 1: 5’-CCGGACCCTGAGGAAGAAGATGTTTCAAGAGAACATCTTCTTCCTCAGGGTTTTTTTG-3’
Oligo 2: 5’-AATTCAAAAAAACCCTGAGGAAGAAGATGTTCTCTTGAAACATCTTCTTCCTCAGGGT-3’
Shβ-catenin -2 Oligo 1: 5’-CCGGCTGTTGTGGTTAAACTCCTTTCAAGAGAAGGAGTTTAACCACAACAGTTTTTTG-3’
Oligo 2: 5’-AATTCAAAAAACTGTTGTGGTTAAACTCCTTCTCTTGAAAGGAGTTTAACCACAACAG-3’
Shβ-catenin -3 Oligo 1: 5’-CCGGGAAGATGTTGACACCTCCCTTCAAGAGAGGGAGGTGTCAACATCTTCTTTTTTG-3’
Oligo 2: 5’-AATTCAAAAAAGAAGATGTTGACACCTCCCTCTCTTGAAGGGAGGTGTCAACATCTTC-3’
Shβ-catenin -4 Oligo 1: 5’-CCGGGGACAAGCCACAGGATTACAACTCGAGTTGTAATCCTGTGGCTTGTCCTTTTTTG-3’
Oligo 2: 5’-AATTCAAAAAAGGACAAGCCACAGGATTACAACTCGAGTTGTAATCCTGTGGCTTGTCC -3’
Shβ-catenin -5 Oligo 1: 5’-CCGGGCGTTATCAAACCCTAGCCTTCTCGAGAAGGCTAGGGTTTGATAACGCTTTTTTG-3’
Oligo 2: 5’-AATTCAAAAAAGCGTTATCAAACCCTAGCCTTCTCGAGAAGGCTAGGGTTTGATAACGC-3’
NC Oligo 1: 5’-CCGGTTCTCCGAACGTGTCACGTTTCAAGAGAACGTGACACGTTCGGAGAATTTTTG-3’
Oligo 2: 5’- AATTCAAAAAATTCTCCGAACGTGTCACGTTCTCTTGAAACGTGACACGTTCGGAGAA-3’

### 2.2. Cell Culture and Administration

MLE-12 cell line was purchased from ATCC and grown in equal parts DMEM and F12 medium (Gibco, Grand Island, NY, USA) with 10% FBS (Gibco) at 37 °C, 5% CO_2_. MLE-12 cells were plated in 6-well plates (2 × 10^6^ cells/well), and the Lv-sh-β-catenin -1, -2, -3, -4, -5 (KD) and NC were incubated with cells at the multiplicity of infection (MOI = 50) for 8 h. After this, the medium was replaced with fresh medium without FBS and the incubation was continued for another 72 h. Cells of each group were given serum-free medium containing 200 μg/mL of silica, and incubated for 18 h [15]. Total mRNA and proteins were extracted for use in real-time PCR and western blot respectively.

After MLE-12 cells infected with Lv-shβ-catenin or Lv-shβ-catenin-NC, cell viability was measured using a MTT Cell Viability Assay Kit (Abnova, Taiwan, China) according to the manufacturer’s protocol. To detect anti-fibrogenic role by Lv-shβ-catenin in MLE-12 cells that had been treated by silica, normal MLE-12 cells, MLE-12 infected with Lv-shβ-catenin, or MLE-12 cells infected with Lv-shβ-catenin-NC were plated at a density of 1 × 10^6^ cells/mL in serum-free DMEM and F12 medium in 6-well plates. After 2 h at 37 °C, cells were given serum-free medium containing 200 μg/mL of silica, and incubated for 18 h [15]. Then supernatants and cells were collected for further experiments.

### 2.3. Experimental Animals and Design

Equal proportions of male and female C57BL/6 mice at 6–8 weeks of age, weighing 16–18 g, were obtain from SLAC Laboratory Animal Co. Ltd. (Shanghai, China). The permit number of animal Care and Use Committee of China Medical University is CMU62043013. The animals were housed at an environmental temperature of 24 ± 1 °C and a 12/12 h light/dark cycle, with free access to food and water. SiO_2_ was purchased from U.S. Silica Company (Frederick, MD, USA). The silica content of the SiO_2_ was > 99%, the dust particle size distribution: 97% < 5 μm diameter, 80% < 3 μm diameter. All experiments and surgical procedures were approved by the Animal Care and Use Committee at the China Medical University, which complies with the National Institute of Health Guide for the Care and Use of Laboratory Animals.

Animals were divided randomly into the following four experimental groups (n = 18 per group): (1) saline control group: instillation of 0.1 mL of sterile physiological saline; (2) silica group: instillation of a suspension of 1 mg silica dust in a total volume of 0.1 mL sterile physiological saline; (3) silica + Lv-shβ-catenin group: instillation of a mixed suspension of 1 mg of silica dust and 5 × 10^7^ transducing units (TU) of Lv-shβ-catenin in a total volume of 0.1 mL of sterile physiological saline; (4) silica + Lv-shβ-catenin-NC group: instillation of a mixed suspension of 1 mg of silica dust and 5 × 10^7^ TU Lv-shβ-catenin-NC in a total volume of 0.1 mL of sterile physiological saline. Mice were anesthetized with an intraperitoneal injection of 0.1 mL/mouse with 10% chloral hydrate. The skin of the neck was opened and blunt dissection exposed the trachea. Either physiological saline, silica in physiological saline, or silica with Lv-shβ-catenin or Lv-shβ-catenin-NC in physiological saline, was instilled into the lungs using a 7-gauge needle inserted into the trachea through the epiglottis of the larynx. The site of surgery was sutured and the mice were allowed to recover until they were sacrificed. At 7, 28 and 56 days post-instillation, six mice of each group were anesthetized with anesthetic ether, sacrificed by decapitation, and the lungs were removed. Bronchoalveolar lavage fluid (BALF) was obtained by cannulating the trachea, injecting and retrieving 1 mL aliquots of sterile physiological saline. Then BALF was centrifuged at 1000 rpm for 1 min at 4 °C, and stored at –80 °C.

### 2.4. Quantitative Real-Time PCR Analysis

Quantitative real-time PCR (qRT-PCR) analysis was performed as previously described [1]. Briefly, total RNA was isolated from MLE-12 cells using the TRIZOL Reagent (Invitrogen, Carlsbad, CA, USA) according to the manufacturer’s protocol. The RNA concentration and the ratio of A 260/280 of were determined by UV spectrophotometry. Five μg of total RNA of each sample was reverse transcribed in a volume of 0.1 mL using a reverse transcription kit (Takara, Otsu, Shiga, Japan) following the manufacturer’s instructions. Primers were designed with the Primer 3 [16] and the specificity of sequences was verified by BLAST program [17]. PrimeScript RT-PCR kit (Takara, Otsu, Shiga, Japan) was used for qRT-PCR. The primer sequences were as follows: β-catenin, sense: 5’-ATGGAGCCGGACAGAAAAGC-3’, antisense: 5’-CTTGCCACTCAGGGAAGGA-3’; MMP2, sense: 5’-CAAGTTCCCCGGCGATGTC-3’, antisense: 5’-TTCTGGTCAAGGTCACCTGTC-3’; MMP9, sense: 5’-CTGGACAGCCAGACACTAAAG-3’, antisense: 5’-CTCGCGGCAAGTCTTCAGAG-3’; GAPDH, sense: 5’-GGTTGTCTCCTGCGACTTCA-3’, antisense: 5’-CCACCACCCTGTTGCTGTAG-3’. Each PCR reaction mixture (20 μL) contained 10 μL of 2 × SYBR Green Master Mix (Takara), 1 μL of forward and reverse primers (5 µmol/μL), 1 µL of cDNA product and supplement water up to 20 μL. The PCR reactions were run on ABI 7500 (Applied Biosystems, Foster City, CA, USA) using the following program: 95 °C for 15 s, followed by 45 cycles of 95 °C for 5 s and 60 °C for 30 s. Analysis was performed using the 7500 system software (Applied Biosystems).

### 2.5. Western Blot Analysis

For western blot analysis, total protein was extracted from MLE-12 cells and lung homogenates with M-PER and T-PER Protein Extraction Reagents (Thermo, Rockford, IL, USA), respectively which containing 0.1 mM of phenylmethyl sulfonyl fluoride (Sigma, St. Louis, MO, USA) according to manufacturer’s protocol. 20–40 µg proteins were separated by 10% SDS-PAGE under a constant voltage of 120 V for 2 h and then transferred to a nitrocellulose membrane at a constant voltage of 300 V for 1 h using a Mini-PROTEAN tetra system (Bio-Rad, Hercules, CA, USA). After blocking with phosphate buffer saline containing 0.05% Tween-20 (PBST) and 5% non-fat dry milk, the membranes were incubated at 4 °C overnight with TBST and 5% milk containing primary antibodies at following dilution ratios: anti-β-catenin antibody (1:2000, Cell Signaling Technology, Beverly, MA, USA); anti-MMP9 antibody (1:500, Proteintech, Chicago, IL, USA); anti-MMP2 antibody (1:500, Proteintech) and anti-GAPDH antibody (1:2000, Cell Signaling Technology, Beverly, MA, USA). The membranes were then washed three times with PBST and incubated with horseradish peroxidase-conjugated anti-rabbit IgG, HRP-linked antibody (1:5000, Cell Signaling Technology) for 1 h at room temperature (RT), followed by washing with PBST. Proteins were visualized by chemiluminescence (ECL, Amersham Biosciences, GE Healthcare, UK).

### 2.6. ELISA Assay for TGF-β1

The ELISA plate was coated with 100 µL of capture antibody in coating buffer per well of ELISA kit (eBioscience, San Diego, CA, USA) and incubated overnight at 4 °C. The plate was washed with 250 µL of wash buffer. Then the well was blocked with 200 µL of assay diluent, incubated 1 h at RT. A volume of 100 µL cell supernatants or BALF or the different dilutions of standard (for standard curve) was added to each well, incubated 2 h at RT. The well was incubated at RT with 100 µL detection antibody 1 h, followed by 100 µL of avidin-HRP for 30 min and 100 µL of substrate solution for 15 min. Before plate reading at 450 nm, 50 µL of Stop Solution was added. The ELISA assay was performed in triplicate.

### 2.7. Determination of Hydroxyproline Content

The lung samples were measured for hydroxyproline content using a hydroxyproline kit from Nanjing Jian Cheng Institute (Nanjing, China) following instruction from the manufacturer. The results were calculated as milligrams of hydroxyproline per gram of wet lung weight.

### 2.8. Pathological Examination

Following gross inspection of each mouse, small pieces of lung tissue from the middle of the lobes, along with the hilar lymph nodes, were fixed with 4% paraformaldehyde, embedded in paraffin, and sectioned at 5 µm. The tissue sections were stained with hematoxylin and eosin (HE). Silicotic nodules were graded as following: cellular nodules as Stage I; fibrotic cellular nodules as Stage II; cellular fibrotic nodules as Stage III; fibrotic nodules as Stage IV [16].

### 2.9. Immunohistochemical Staining

For immunohistochemical examination, all sections were deparaffinized in xylene followed by 100% ethanol and then placed in a freshly prepared methanol plus 3% H_2_O_2_ solution for 30 min to block endogenous peroxidase activity. After overnight incubation at 4 °C with rabbit polyclonal anti-collagen I and III antibodies (1:100, Santa Cruz Biotechnology, Santa Cruz, CA, USA), anti-MMP9 antibody (1:100, Proteintech), anti-MMP2 antibody (1:100, Proteintech) diluted in phosphate-buffered saline (PBS), antigen-antibody complexes were detected using Streptavidin/Peroxidase (SP) Histostain^TM^-Plus Kits (Beijing Zhongshan Golden Bridge Biotechnology Ltd., Beijing, China). Peroxidase activity was revealed using a 3,3’-diaminobenzidine tetrahydrochloride Substrate Kit (Beijing Zhongshan Golden Bridge Biotechnology Ltd.). The sections were counterstained with hematoxylin for 3 min, rinsed and mounted with glycerin gelatin for histological examination. Brown particles in the cytoplasm or the cellular membrane were considered a positive reaction. The collagen I, collagen III, MMP2 and MMP9 proteins were analyzed quantitatively using MetaMorph/DP10/BX41-type image analysis software (UIC/Olympus, Chicago, IL, USA/ Tokyo, JP). In 10 × 40 fields, three to five fields were randomly selected for each section. The integrated optical density (IOD) average represented the quantitative expression of collagen I, collagen III, MMP2 and MMP9.

### 2.10. Statistical Analyses

SPSS 17.0 software was used to conduct statistical analyses. The differences between values were evaluated through one-way analysis of variance (ANOVA) followed by pair-wise comparison with the Student-Newman-Keuls test. *p* < 0.05 was considered statistically significant.

## 3. Results

### 3.1. Construction and Screening of Lv-shβ-Catenin Vectors

**Figure 1:**
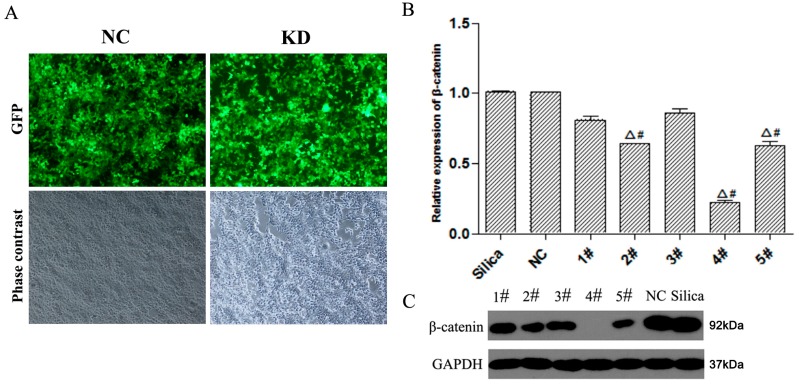
Infection of MLE-12 cells with non-silenced control (NC) or Lv-shβ-catenin (KD). (**A**) Cells were infected with NC or Lv-shβ-catenin. Phase contrast image and GFP expression under a fluorescent microscope was taken after 72 h. (**B**) After MLE-12 cells were treated with Lv-shβ-catenin, mRNA level of β-catenin was detected by realtime-PCR by using -_ΔΔ_ Ct method. Data are mean ± SEM (n = 3). (**C**) Protein level of β-catenin was detected by western blot. Cells of each group were incubated with silica. Lanes 1–5: Lv-shβ-catenin-1, -2, -3, -4, -5, respectively. ^Δ^ *p* < 0.05, compared to the silica group; ^#^ *p* < 0.05, compared to the NC group.

MLE-12 cells were infected with Lv-shβ-catenin -1, -2, -3, -4,-5, or -NC, respectively. Cell viability was above 80% of control at 24 to 96 h of virus infection (data not shown). After 72 h cell image was taken by fluorescence microscopy. We observed the expression of green fluorescent protein in a large number of MLE-12 cells, which indicates a decent infection effect by Lv-shβ-catenin (KD) and Lv-shβ-catenin-NC (NC) (Figure 1A). The expression of β-catenin was increased in silica-treated MLE-12 cells (Supplemental Information, Figure S1). Real-time PCR was then used to determine the level of β-catenin mRNA in MLE-12 cells. Infection with Lv-shβ-catenin-1, -2, -3, -4 or -5 suppressed β-catenin mRNA expression by approximately 19%, 37%, 14%, 78% or 38%, respectively, compared to Lv-shβ-catenin-NC. These results demonstrated that Lv-shβ-catenin-4 is most effective in suppressing β-catenin mRNA expression in the MLE-12 cells (Figure 1B). Western blot analysis also demonstrated that infection with Lv-shβ-catenin-1, -2, -3, -4 or -5 suppressed β-catenin protein expression in MLE-12 cells. Moreover the silencing effect of Lv-shβ-catenin-4 was most obvious. (Figure 1C). Thus Lv-shβ-catenin-4 was used as the Lv-shβ-catenin in subsequent experiments.

### 3.2. Effect of Lv-shβ-Catenin on Silica-Treated MLE-12 Cells

**Figure 2:**
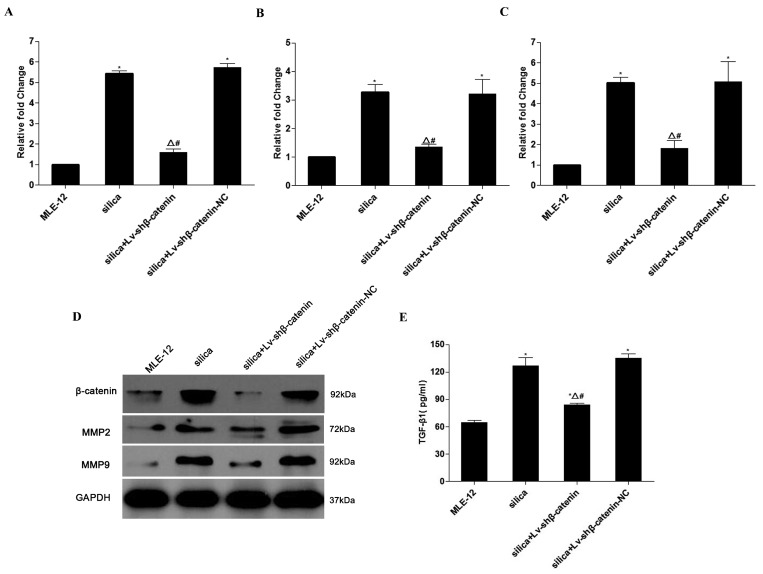
Effects of Lv-shβ-catenin on expression of β-catenin, MMP2, and MMP9, and secretion of TGF-β1 in the silica-treated MLE-12 cells. (**A**) mRNA level of β-catenin. (**B**) mRNA level of MMP2. (**C**) mRNA level of MMP9. (**D**) Protein level of β-catenin, MMP2 and MMP9 in silica-treated MLE-12 cells assayed by western blot. (**E**) Level of TGF-β1 in the supernatants of silica-treated MLE-12 cells determined by ELISA. Data are mean ± SEM (n = 3). ^*^ *p* < 0.05, compared to the MLE-12 group; ^Δ^ *p* < 0.05, compared to the silica group; ^#^ *p* < 0.05, compared to the silica + Lv-shβ-catenin-NC group.

To examine the effects of Lv-shβ-catenin on expression of β-catenin, MMP2, and MMP9, and secretion of TGF-β1 in the silica-treated MLE-12 cells, real-time PCR, western blot and ELISA assays were performed (Figure 2).The results showed that Lv-shβ-catenin significantly suppressed β-catenin mRNA and protein expression in cells activated by silica (Figure 2A,D). As MMPs play important roles in the pathogenesis of silicosis, we examined the expression of MMP2 and MMP9 in silica-treated MLE-12 cells. Lv-shβ-catenin significantly reduced the expression of MMP2 (*p* < 0.05) (Figure 2B,D) and MMP9 (*p* < 0.05) (Figure 2C,D) insilica-treated MLE-12 cells. An analysis of TGF-β1 in cell supernatants indicated that treatment with Lv-shβ-catenin significantly inhibited TGF-β1 secretion (*p* < 0.05) (Figure 2E).

### 3.3. Inhibition of β-Catenin Expression by Lv-shβ-Catenin in Vivo

**Figure 3:**
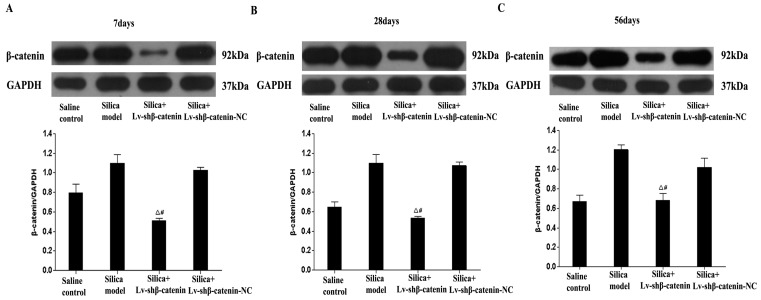
Expression of β-catenin in the lung of silica-administrated mice at (**A**) 7 days, (**B**) At 28 days and (**C**) 56 days after the instillation. Data are expressed as mean ± SE (n = 3). ^Δ^ *p* < 0.05, compared to the silica group; ^#^ *p* < 0.05, compared to the silica + Lv-shβ-catenin-NC group.

We used western blot assays to examine the silencing effects of *Lv-shβ-catenin* in the lung of silica—administrated mice. The results demonstrate that expression of β-catenin mRNA in the silica+Lv-shβ-catenin group was significantly lower than in the saline control, silica, and silica+Lv-shβ-catenin-NC groups (*p* < 0.05) at the three time points after instillation (Figure 3).

### 3.4. Inhibition of Silica-Induced Lung Fibrosis by Lv-shβ-Catenin in Vivo

#### 3.4.1. Lv-shβ-Catenin Could Reduce TGF-β1 Content in BALF

**Figure 4:**
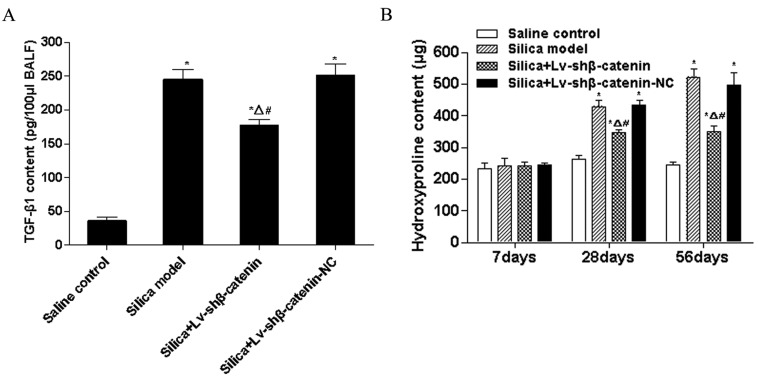
TGF-β1 level in BALF and hydroxyproline content in the lung of mice instilled with silica. (**A**) The level of TGF-β1 in BALF detected by ELISA at 7 days after silica instillation. (**B**) Hydroxyproline content in the lung of mice at different time point after silica instillation. Data are mean ± SEM (n = 6). ^*^ *p* < 0.05, compared to the saline control group; ^Δ^ *p* < 0.05, compared to the silica group; ^#^ *p* < 0.05, compared to the silica + Lv-shβ-catenin-NC group.

TGF-β1 plays a critical role in the progression of lung fibrosis. In this study, TGF-β1 contents of the silica group and the silica + Lv-shβ-catenin-NC group were significantly higher than those of the saline control group (*p* < 0.05) at 7 days after instillation. TGF-β1 content of the silica + Lv-shβ-catenin group was significantly lower than in the silica group and the silica + Lv-shβ-catenin-NC group (*p* < 0.05), and significantly higher than that of the saline control group (*p* < 0.05) at 7 days after instillation (Figure 4A).

#### 3.4.2. Lv-shβ-Catenin Could Reduce Hydroxyproline Content in the Lung

Hydroxyproline content is an important indicator of lung fibrosis. In this study, no significant differences in the hydroxyproline content of the four treatment groups were observed at 7 days after instillation when measured using a hydroxyproline kit.

However, hydroxyproline content of the silica group and the silica + Lv-shβ-catenin-NC group was significantly higher than those of the saline control group (*p* < 0.05) at 28 and 56 days after instillation. Hydroxyproline content of the silica + Lv-shβ-catenin group was significantly lower than that of the silica group and the silica + Lv-shβ-catenin-NC group (*p* < 0.05), and significantly higher than that of the saline control group (*p* < 0.05) at 28 and 56 days after instillation (Figure 4B).

#### 3.4.3. Lv-shβ-Catenin Could Alleviate Histopathologic Changes in the Lung

**Figure 5:**
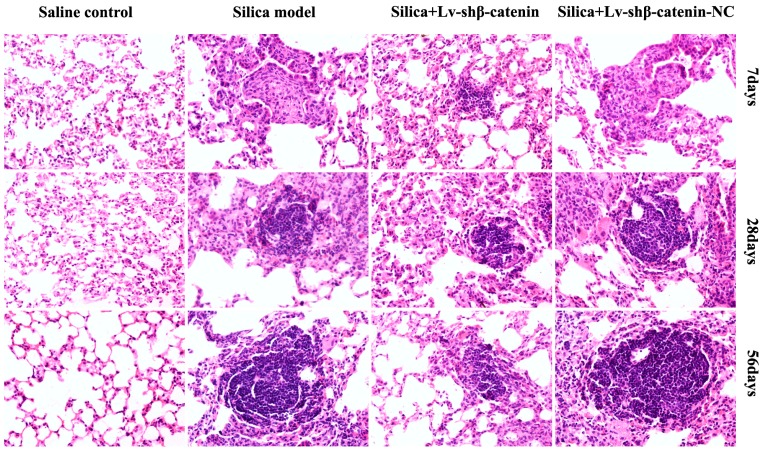
HE staining for histopathologic changes in mice lungs (×400).

Lung tissues of mice were observed by light microscope to monitor pathological changes. No obvious abnormalities were observed in the lung of mice that received physiological saline. However, in the silica and silica + Lv-shβ-catenin-NC groups, there was a large infiltration of inflammatory cells and alveolar septal thickening in the lung, and occasionally a small amount of cellular nodules (Stage I) were observed at 7 days after instillation. There were less cellular nodules (Stage I) in the lung of mice in the silica + Lv-shβ-catenin group. At 28 days after instillation, primarily cellular nodules and fibrotic cellular nodules (Stage I+ and II) were observed in the silica and the silica + Lv-shβ-catenin-NC groups. There were mainly cellular nodules (Stage I) in the lung of mice in the silica + Lv-shβ-catenin group. Compared to the silica and the silica + Lv-shβ-catenin-NC groups, nodules in the lung of mice in the silica + Lv-shβ-catenin group was less in count and smaller in size. In the silica and the silica + Lv-shβ-catenin-NC groups, fibrotic cellular nodules (Stage II+ and III) were observed at 56 days after the instillation. There were still mostly cellular nodules (Stage I+) and fibrotic cellular nodules (Stage II) in the lung of mice from the silica + Lv-shβ-catenin group, and the number of nodules was increased (Figure 5 and Table 2).

**Table 2:** Silicotic nodule grade of the mouse lungs in each group.

Groups	7 Days After Instillation		28 Days After Instillation		56 Days After Instillation	
	n	Silicotic Nodule Grade	n	Silicotic Nodule Grade	n	Silicotic Nodule Grade
Saline control	6	0	6	0	6	0
Silica	6	I	6	I+ ~ II	6	II+ ~ III
Silica + Lv-shβ-catenin	6	0 ~ I	6	I ~ I+	6	I+ ~ II
Silica + Lv-shβ-catenin-NC	6	I	6	I+ ~ II	6	II+ ~ III

#### 3.4.4. Lv-shβ-Catenin Could Inhibit the Expression of Collagen I, Collagen III, MMP2 and MMP9 in the Lung

To further observe the degree of fibrosis in the lung, immunohistochemical examination of collagen I, collagen III, MMP2 and MMP9 was performed. Representative images are shown in Supplemental Information, Figure S2. A weakly positive reaction for scattered collagen I was observed in the mesenchymal tissue of the saline control group. At the three time points after the instillation, the IOD average of collagen I in the silica and the silica + Lv-shβ-catenin-NC groups was significantly higher than that of the saline control groups (*p* < 0.05).

**Figure 6:**
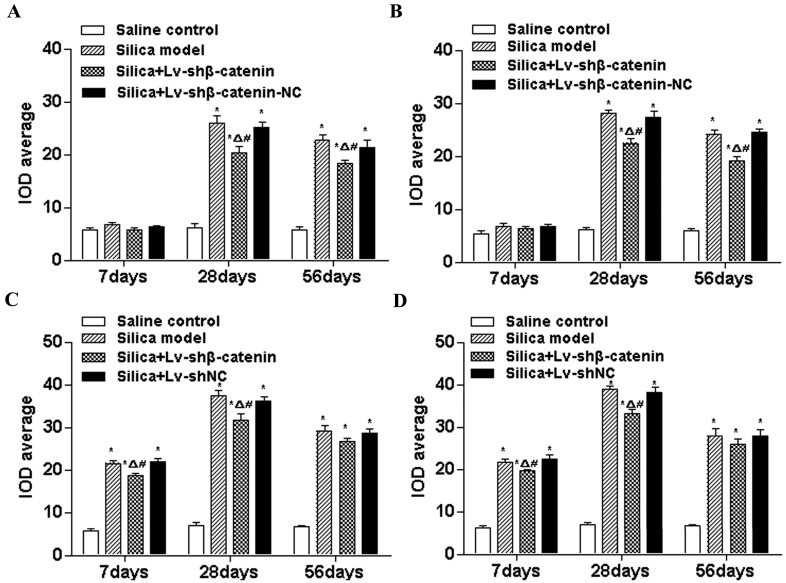
Average IOD of collagen I (**A**), collagen III (**B**), MMP2 (**C**) and MMP9 (**D**) in the lung of mice at different time points after silica-administration. Data are expressed as mean ± SEM. (n = 6). ^*^ *p* < 0.05, compared to the saline control group; ^Δ^ *p* < 0.05, compared to the silica group; ^#^ *p* < 0.05, compared to the silica + Lv-shβ-catenin-NC group.

At 28 days and 56 days after instillation, the IOD average of collagen I in the silica + Lv-shβ-catenin group was higher than that of the saline control groups (*p* < 0.05), but was significantly lower than that of the silica and silica + Lv-shβ-catenin-NC groups (*p* < 0.05) (Figure 6A). Alterations of collagen III were concordant with those of collagen I (Figure 6B). At 7 days and 28 days after instillation, the IOD average of MMP2 in the silica + Lv-shβ-catenin group was higher than that of the saline control group (*p* < 0.05), but was significantly lower than that of the silica and silica + Lv-shβ-catenin-NC groups (*p* < 0.05) (Figure 6C). Alterations of MMP9 were similar to those of collagen I (Figure 6D).

## 4. Discussion

Pulmonary fibrosis is the most important pathological change in silicosis. Although the pathogenesis of silicosis has not been clearly explained, recent studies have suggested that the Wnt/β-catenin signaling pathway is involved in the development of pulmonary fibrosis [6,15]. Following silica-induced lung injury, the Wnt/β-catenin signaling pathway is abnormally activated, which leads to upregulation of β-catenin expression. Typically, Wnt ligand binds to the cell surface receptor frizzelds. Then Wnt is disheveled in the cytoplasm and recruited to the cell membrane, which promotes the phosphorylation of glycogen synthase kinase-3β and thus inhibits the phosphorylation of β-catenin. The phosphorylated β-catenin can not be recognized by ubiquitin and thus fails to be degraded, resulting in a large number of free β-catenin aggregating in the cytoplasm. This breaks the balance of β-catenin between the cytoplasm and nucleus, increases the content of nuclear β-catenin, and ultimately initiates transcription of the downsteam genes that are involved in lung injury and fibrosis [18,19].

Accordingly, elevated expression of β-catenin plays a key role in the Wnt/β-catenin signaling pathway. To investigate whether inhibition of β-catenin expression attenuates lung fibrosis, we used RNAi technology to construct a recombinant lentiviral vector, Lv-shβ-catenin, which expressed shRNA specific against mouse β-catenin. We confirmed the β-catenin-silencing efficacy of shβ-catenin in two different cell lines, MLE-12 and HEK-293T cells (data not shown), as well as in the mouse model of silicosis. The results showed that a recombinant shβ-catenin lentivirus was successfully constructed, which could interfere with the expression of β-catenin both *in vitro* and *in vivo*.

MMP2 and MMP9 are expressed in many types of cells in the lung tissue, such as bronchiolar epithelial cells, fibroblasts, macrophages, and so on [20]. These MMPs are important target genes controlled by the Wnt/β-catenin signaling pathway [21,22]. In the development of pulmonary fibrosis, MMPs specifically cleave important constituents of extracellular matrix (ECM) such as gelatin and collagen, and therefore damage the structure of alveolar wall [20]. Our *in vitro* and *in vivo* experiments showed that silencing β-catenin suppressed the expression of MMP2 and MMP9 at both mRNA and protein levels, which provide a possible mechanism underlying the role of Wnt/β-catenin signaling pathway in lung fibrosis. The cytokine TGF-β1 also contributes to the progression of lung fibrosis [23,24]. We found that inhibition of the Wnt/β-catenin signaling pathway by Lv-shβ-catenin could suppress the secretion of TGF-β1 by silica-treated MLE-12 cells, and also in BALF of silicosis mice. This indicates that TGF-β1 secretion is regulated, at least in part, by the Wnt/β-catenin signaling pathway.

An experimental mouse model of silicosis was induced by an intratracheal administration of silica dust in the present study. This method was proved to result in varying degrees of fibrotic silicosis [25]. Lv-shβ-catenin effectively suppressed the elevated expression of β-catenin in the lung of silica-administrated mice. More importantly, lung fibrosis was alleviated by Lv-shβ-catenin as evidenced by HE staining, hydroxyproline content and collagen accumulation in the lung. In accordance with results *in vitro*, attenuation of MMPs expression in the lung and TGF-β1 secretion in BALF by Lv-shβ-catenin was observed in mice of silicosis, however, presented earlier than onset of alteration in lung fibrosis (7 days *versus* 28 days and 56 days after silica instillation), which suggests the role of MMPs and TGF-β1 in the development of lung fibrosis. Therefore, we may conclude that Lv-shβ-catenin could prevent the development of silica-induced lung fibrosis. This study provides a new molecular basis for understanding the mechanism of silicosis. The Wnt/β-catenin signaling pathway may represent a novel target for prevention and treatment of silicosis.

## 5. Conclusions

Our findings suggest that blockade of the Wnt/β-catenin signaling pathway by silencing β-catenin prevents the development of silica-induced lung fibrosis, which is related to the expression of MMP2 and MMP9, and secretion of TGF-β1 in the lung. Our data support the concept that the Wnt/β-catenin signaling pathway may represent a novel target for prevention and treatment of silicosis.

## Supplementary Files

Supplementary File 1

## References

[1] Liu F., Liu J., Weng D., Chen Y., Song L., He Q., Chen J. (2010). CD4+CD25+Foxp3+ regulatory T cells depletion may attenuate the development of silica-induced lung fibrosis in mice. PLoS ONE.

[2] Song L., Weng D., Liu F., Chen Y., Li C., Dong L., Tang W., Chen J. (2012). Tregs promote the differentiation of Th17 cells in silica-induced lung fibrosis in mice. PLoS ONE.

[3] Huang C., Ma R., Xu Y., Li N., Li Z., Yue J., Li H., Guo Y., Qi D. (2015). Wnt2 promotes non-small cell lung cancer progression by activating WNT/β-catenin pathway. Am. J. Cancer Res..

[4] Kim T.H., Kim S.H., Seo J.Y., Chung H., Kwak H.J., Lee S.K., Yoon H.J., Shin D.H., Park S.S., Sohn J.W. (2011). Blockade of the Wnt/β-catenin pathway attenuates bleomycin-induced pulmonary fibrosis. Tohoku J. Exp. Med..

[5] Königshoff M., Balsara N., Pfaff E.M., Kramer M., Chrobak I., Seeger W., Eickelberg O. (2008). Functional Wnt signaling is increased in idiopathic pulmonary fibrosis. PLoS ONE.

[6] Liu L., Carron B., Yee H.T., Yie T.A., Hajjou M., Rom W. (2009). Wnt pathway in pulmonary fibrosis in the bleomycin mouse model. J. Environ. Pathol. Toxicol. Oncol..

[7] Chilosi M., Poletti V., Zamò A., Lestani M., Montagna L., Piccoli P., Pedron S., Bertaso M., Scarpa A., Murer B. (2003). Aberrant Wnt/beta-catenin pathway activation in idiopathic pulmonary fibrosis. Am. J. Pathol..

[8] Wang Y., Sun Z., Qiu X., Li Y., Qin J., Han X. (2009). Roles of Wnt/beta-catenin signaling in epithelial differentiation of mesenchymal stem cells. Biochem. Biophys. Res. Commun..

[9] Pardo A., Pérez-Ramos J., Segura-Valdez L., Ramírez R., Selman M. (1999). Expression and localization of TIMP-1, TIMP-2, MMP-13, MMP-2, and MMP-9 in early and advanced experimental lung silicosis. Ann. NY Acad. Sci..

[10] Li X.F., Liao J., Xin Z.Q., Lu W.Q., Liu A.L. (2013). Relaxin attenuates silica-induced pulmonary fibrosis by regulating collagen type I and MMP-2. Int. Immunopharmacol..

[11] Cutroneo K.R., White S.L., Phan S.H., Ehrlich H.P. (2007). Therapies for bleomycin induced lung fibrosis through regulation of TGF-beta1 induced collagen gene expression. J. Cell Physiol..

[12] Henderson W.R., Chi E.Y., Ye X., Nguyen C., Tien Y.T., Zhou B., Borok Z., Knight D.A., Kahn M. (2010). Inhibition of Wnt/beta-catenin/CREB binding protein (CBP) signaling reverses pulmonary fibrosis. Proc. Natl. Acad Sci. USA..

[13] Pullmann R., Juhaszova M., de Silanes I.L., Kawai T., Mazan-Mamczarz K., Halushka M.K., Gorospe M. (2005). Enhanced proliferation of cultured human vascular smooth muscle cells linked to increased function of RNA-binding protein HuR. J. Biol. Chem..

[14] Sakoda T., Kasahara N., Hamamori Y., Kedes L. (1999). A high-titer lentiviral production system mediates efficient transduction of differentiated cells including beating cardiac myocytes. J. Mol. Cell Cardiol..

[15] Wang X., Chen Y., Lv L., Chen J. (2009). Silencing CD36 gene expression results in the inhibition of latent-TGF-β1 activation and suppression of silica-induced lung fibrosis in the rat. Respir. Res..

[16] Free Online Primer Design Tool. http://frodo.wi.mit.edu/primer3.

[17] Basic Local Alignment Search Tool. http://www.blast.ncbi.nlm.nih.gov/.

[18] Cohen J.C., Larson J.E., Killeen E., Love D., Takemaru K. (2008). CFTR and Wnt/beta-catenin signaling in lung development. BMC Dev. Biol..

[19] Wang Z., Zhang J.N., Hu X.F., Chen X.L., Wang X.R., Zhao T.T., Peng M.J., Zou P. (2010). Effects of pentoxifylline on Wnt/β-catenin signaling in mice chronically exposed to cigarette smoke. Chin. Med. J..

[20] O’Connor C.M., FitzGerald M.X. (1994). Matrix metalloproteases and lung disease. Thorax.

[21] Zuo F., Kaminski N., Eugui E., Allard J., Yakhini Z., Ben-Dor A., Lollini L., Morris D., Kim Y., DeLustro B., Sheppard D., Pardo A., Selman M., Heller R.A. (2002). Gene expression analysis reveals matrilysin as a key regulator of pulmonary fibrosis in mice and humans. Proc. Natl. Acad Sci. USA.

[22] Wu B., Crampton S.P., Hughes C.C. (2007). Wnt signaling induces matrix metalloproteinase expression and regulates T cell transmigration. Immunity.

[23] Gu H., Mickler E.A., Cummings O.W., Sandusky G.E., Weber D.J., Gracon A., Woodruff T., Wilkes D.S., Vittal R. (2014). Crosstalk between TGF-β1 and complement activation augments epithelial injury in pulmonary fibrosis. FASEB J..

[24] Lee C.M., Park J.W., Cho W.K., Zhou Y., Han B., Yoon P.O., Chae J., Elias J.A., Lee C.G. (2014). Modifiers of TGF-β1 effector function as novel therapeutic targets of pulmonary fibrosis. Korean J. Intern. Med..

[25] Hemmati A.A., Nazari Z., Samei M. (2008). A comparative study of grape seed extract and vitamin E effects on silica-induced pulmonary fibrosis in rats. Pulm. Pharmacol. Ther..

