# Supplementary material for: Blocking the Wnt/β-Catenin Pathway by Lentivirus-Mediated Short Hairpin RNA Targeting β-Catenin Gene Suppresses Silica-Induced Lung Fibrosis in Mice
Source: Int J Environ Res Public Health. 2015 Sep 1;12(9):10739–54. doi: 10.3390/ijerph120910739 (PMC4586640; doi:10.3390/ijerph120910739)
Supplement: Supplementary File 1 [file ijerph-12-10739-s001.pdf]

Supplementary Information

Blocking the Wnt/β-Catenin Pathway by Lentivirus-Mediated Short Hairpin RNA Targeting β-Catenin Gene Suppresses Silica-Induced Lung Fibrosis in Mice

---

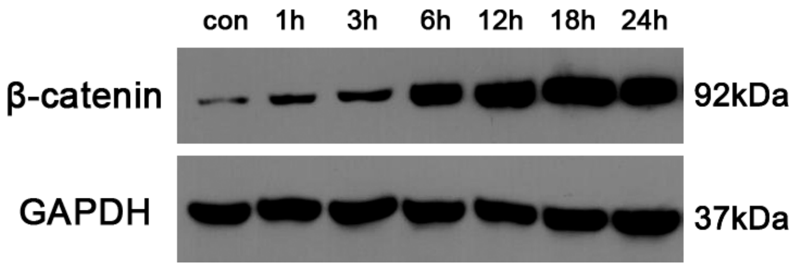

Figure S1. Protein level of β-catenin in the silica-treated MLE-12 cells at each time point.

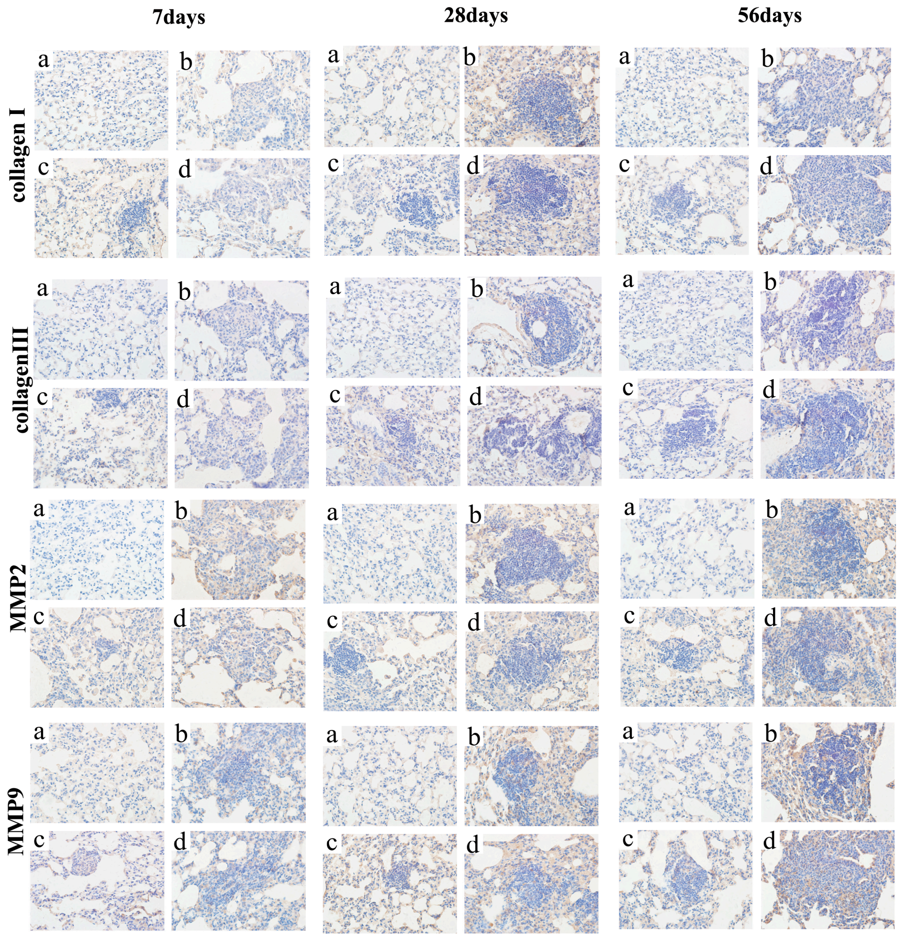

Figure S2. Immunohistochemical staining for collagen I, collagen III, MMP2 and MMP9 at each time point (× 400).

© 2015 by the authors; licensee MDPI, Basel, Switzerland. This article is an open access article distributed under the terms and conditions of the Creative Commons Attribution license (http://creativecommons.org/licenses/by/4.0/).
